# Novel green hydrogen – Fossil fuel dehydrogenation

**DOI:** 10.1016/j.fmre.2024.06.007

**Published:** 2024-06-27

**Authors:** Kaiqiang Zhang, Zhijun Jin, Quanyou Liu, Lirong Liu

**Affiliations:** a Institute of Energy, Peking University, Beijing 100871, China; b Ordos Research Institute of Energy, Peking University, Ordos 017010, China; c Centre for Environment & Sustainability, University of Surrey, Guildford GU2 7XH, United Kingdom

**Keywords:** Hydrogen production, Fossil fuel, Carbon-free process, Techno-economic-environmental analysis, Resource and technology

## Abstract

Climate change requires an immediate transition from fossil fuels to clean energy sources. Although hydrogen is considered a future energy source, over 90% of hydrogen is currently produced from fossil fuels, and large-scale renewable-fed hydrogen processes are limited by current technologies and economic processes. Therefore, hydrogen production from fossil fuels is a significant topic, particularly if fossil fuel-fed hydrogen production and utilization can be absolutely carbon-free. For the first time, this review critically discusses and analyses the current advances and fundamentals of fossil fuel dehydrogenation from the perspective of techno-economic-environmental assessments while considering all potential fossil resources and hydrogen technology. This review concludes that the preference of fossil fuels for any hydrogen production technology is as follows: fossil gas > heavy fossil liquid > light fossil liquid > fossil minerals. Thermo-catalytic hydrocarbon decomposition can outperform most other hydrocarbon reforming and pyrolysis processes owing to its energy efficiency, economic effectiveness, and environmental friendliness. Further, we explore potentially new “green hydrogen” technology and confirm that fossil fuels could be completely decarbonized throughout the full-chain stages from exploration to production and consumption. Overall, this work reveals that fossil fuels can be utilized completely carbon-free and provides technical support for future fossil fuel dehydrogenation and energy decarbonization in academic research, industrial practice, and governmental strategies.

## Nomenclature

**Abbreviations**
API: American Petroleum Institute
APR: aqueous phase reforming
ATR: autothermal reforming
CCS: carbon capture & storage
DBD: dielectric barrier discharge
HPHT: high-pressure high-temperature
IEA: International Energy Agency
IFT: interfacial tension
POX: partial oxidation
PSA: pressure swing adsorption
SARA: saturate, aromatic, resin & asphaltene
SMR: steam methane reforming
WGS: water-gas shift

**Terms**
CH_4_: methane
Co: cobalt
CO_2_: carbon dioxide
Cu: copper
Fe: iron
H2: hydrogen
H_2_O: water
Ir: iridium
Mo: molybdenum
Ni: nickel
Pd: palladium
Pt: platinum
*Re*: rhenium
Rh: rhodium
Ru: ruthenium
W: wolfram

**Units**
°C: Celsius degree
kg: kilogram
m^3^: cubic metre
MPa: mega pascal

## 1 Introduction

Energy and the environment are key components of human society that interact with each other and play important roles in economic development [1]. The undeveloped extraction and storage technologies for renewable energy, along with their lack of cost competitiveness, are limitations [2] in meeting the growing energy demands currently fulfilled by fossil fuels [3]. However, the continuous burning of fossil fuels results in excess greenhouse gas production and worsens climate change, and fossil fuel products have variable costs [4,5]. A report issued by the U.S. Energy Information Administration indicated that by 2040, world energy consumption will increase by 56%. Fossil fuels will occupy 78% of the energy consumption, and the rest will be fulfilled by renewable energy [6]. Thus, energy and environment contradictions must be immediately mitigated for the long-term development of human society, and political standpoints and the global economy should also be considered [[7], [8]].

Numerous studies have attempted to discover a clean and reliable alternative to fossil fuels [[9], [10]]. Hydrogen has long been a promising clean energy carrier for future energy scenarios, which could be beneficial for mitigating climate change and promoting environmental sustainability [[11], [12], [13]]. Hydrogen has a high per-unit mass energy content and can be used as a fuel in various formats, such as direct combustion and storage in fuel cells, without producing greenhouse gas emissions [14,15]. Unlike fossil fuels, hydrogen exists in various forms on Earth; however, hydrogen energy is not readily available in nature [16,17]. Hydrogen production can be categorized into two major domains based on the raw feedstock: fossil fuels and renewables. Over 95% of hydrogen is produced from fossil fuels [18]. Although significant efforts have been made to expand the renewable part as feedstock, its utilization in feeding hydrogen remains low because of underdeveloped technology and elevated costs [19,20]. Hence, to address and mitigate climate change, decarbonizing hydrogen production from fossil fuels (i.e., fossil fuel dehydrogenation) is a realistic approach.

Several widely applied hydrogen production processes, such as steam reforming, auto-reforming, electrolysis, and gasification, have been established for processing the feedstock of fossil fuels. However, each of them has certain advantages and disadvantages [21,22]. For example, steam methane reforming (SMR), partial oxidation (POX), and autothermal reforming (ATR) methods are commonly accepted technologies for large-scale hydrogen production in the industry, but high levels of carbon emissions are generated [23,24]. The catalytic dehydrogenation of fossil fuels exhibits potential superiority in producing hydrogen without accompanying CO_2_ emissions [25,26]. Previous studies have investigated hydrogen production from the thermal pyrolysis of a wide range of hydrocarbons, including methane, hexadecane, diesel, and gasoline fuel. These studies utilized metal- and carbon-based catalysts and were assisted by conventional thermal, plasma, and microwave methods [27,28]. These studies confirmed that catalytic fossil fuel dehydrogenation is a promising method for carbon-free hydrogen production.

In this review, advances in fossil fuel dehydrogenation are critically reviewed and evaluated in terms of hydrogen resources and technologies. All potential fossil resources, including natural gas, crude oil, and fossil minerals, are analysed in terms of their availability and accessibility, properties, recovery technology, cost, applicability, and compatibility as feedstock for hydrogen production. The second part of this review focuses on hydrogen production technologies. The most popular hydrocarbon reforming and pyrolysis processes, including steam, plasma, and aqueous-phase reforming, POX, ATR, and new dehydrogenation technologies, are systematically compared and evaluated. Finally, fossil fuel dehydrogenation is analysed based on the techno-economic-environmental assessments of resources and technology. For the first time, this review comprehensively examines and analyses fossil fuel resources and their hydrogen production technologies from technological, economic, and environmental perspectives. This analysis aims to support future fossil fuel dehydrogenation and energy decarbonization efforts. Additionally, it will be of interest to readers seeking to understand hydrogen production from fossil fuel sources.

## 2 Resources

**Fig. 1:**
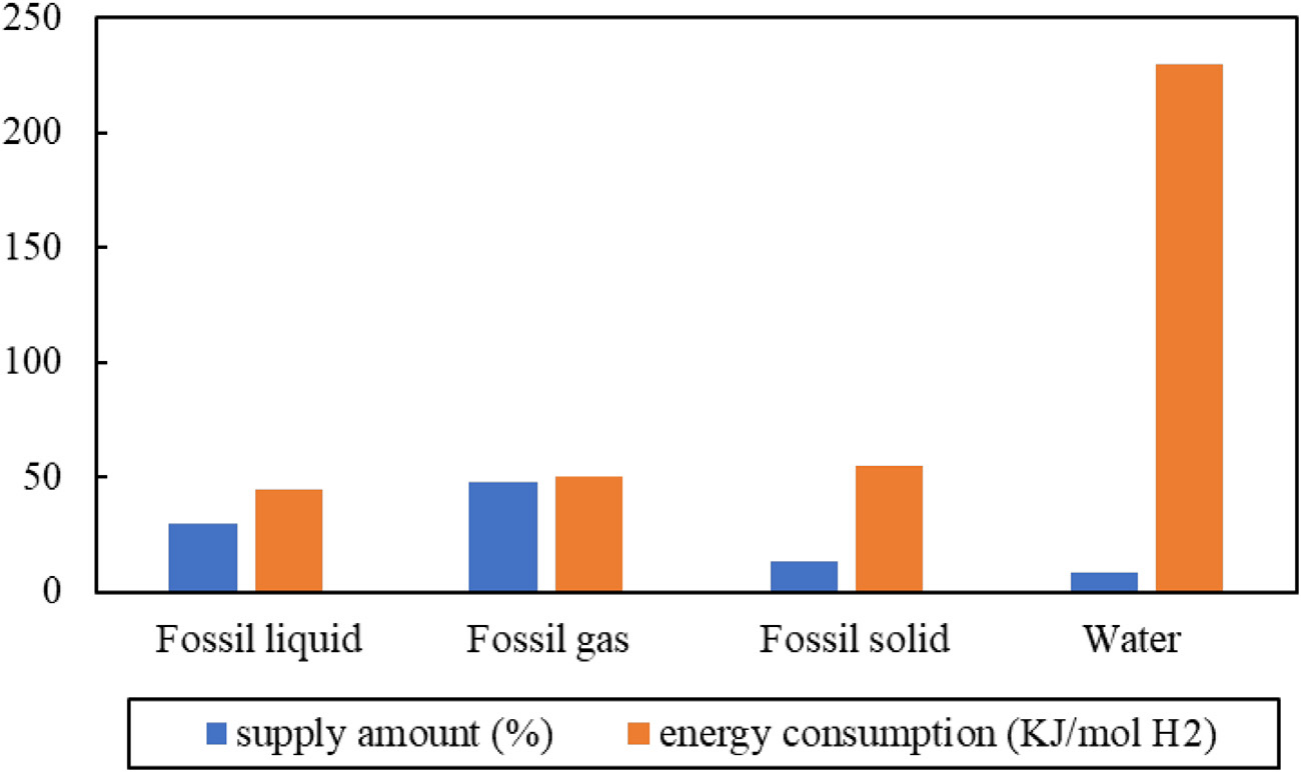
**Hydrogen production resources: supply amount and energy consumption [**[34], [35], [36]**].**

Currently, fossil fuels dominate the global hydrogen supply, with 48% derived from fossil gases (e.g., natural gas), 30% from fossil liquids (e.g., crude oil), and 18% from fossil minerals (e.g., coal) [[29], [30], [31]], as demonstrated in Fig. 1. This figure also illustrates the energy consumption for hydrogen production from fossil fuels and water using current technologies [32,33]. Fossil fuels, particularly fossil liquids and gases, are superior to water because of their consistently large supply and low energy consumption. This paper focuses on fossil-fuel-sourced hydrogen technologies. Fossil fuel resources for hydrogen production are discussed separately from the respective categories of fossil fluids and minerals in the following section.

### 2.1 Fossil fluid

**Fig. 2:**
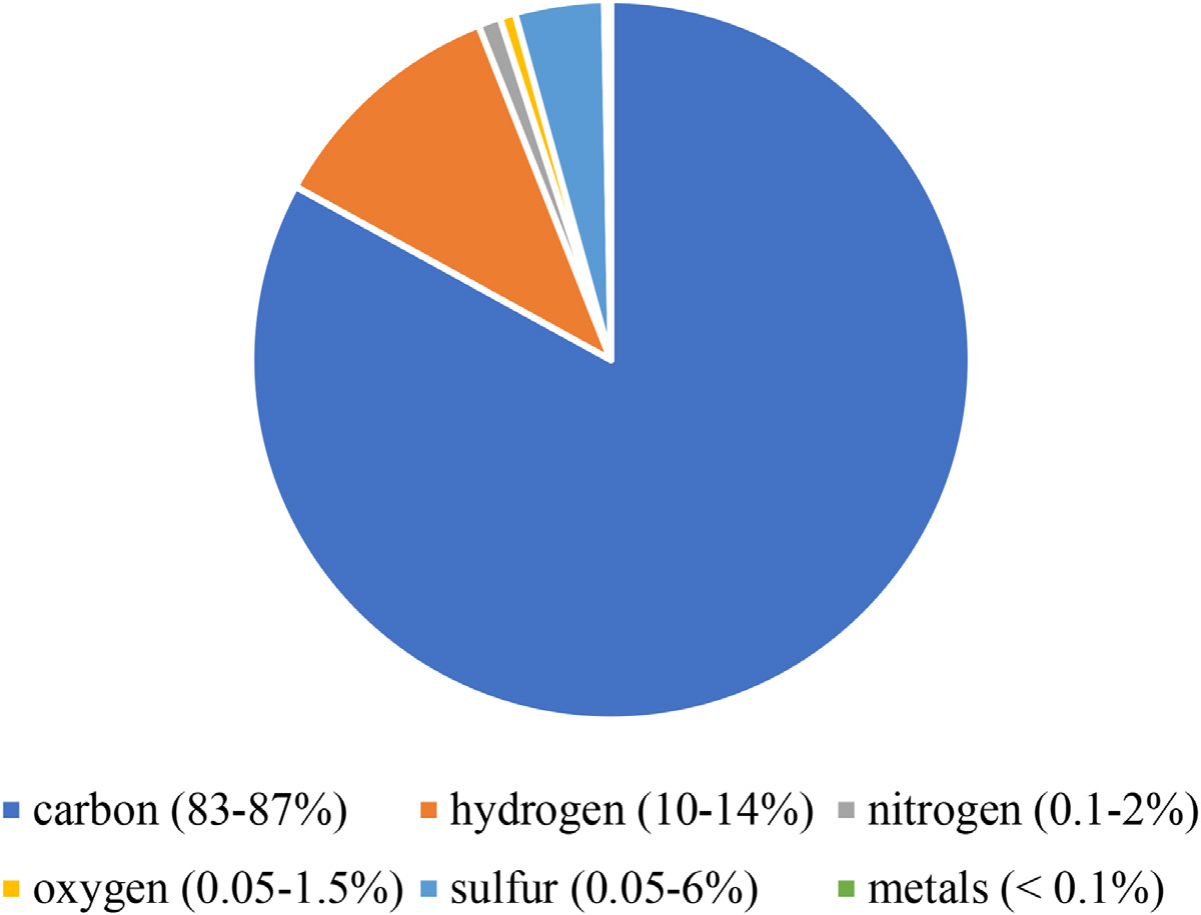
**Typical petroleum fluid compositions [**37**].**

Fossil fluids primarily refer to petroleum fluids (i.e., oil and gas), which are naturally occurring liquefied mixtures that exist in reservoirs under high pressure and temperature conditions. As shown in Fig. 2, petroleum fluids typically include tens of thousands of carbons and a few non-carbons, such as hydrogen, nitrogen, oxygen, sulfide compounds, and metals (e.g., nickel, vanadium, and chromium). Considering the raw material requirements for hydrogen production, fossil fluids are excellent candidates for conversion. However, the properties of fossil fluids depend on the depositional environment of the reservoir from which they are produced. Fossil fluids are typically classified based on their physical properties and chemical constituents. Physical classifications can be easily obtained by measuring various physical properties, such as specific gravity, sulfur, and asphalt content [[37], [38], [39]], whereas chemical properties are more challenging to determine.

**Fig. 3:**
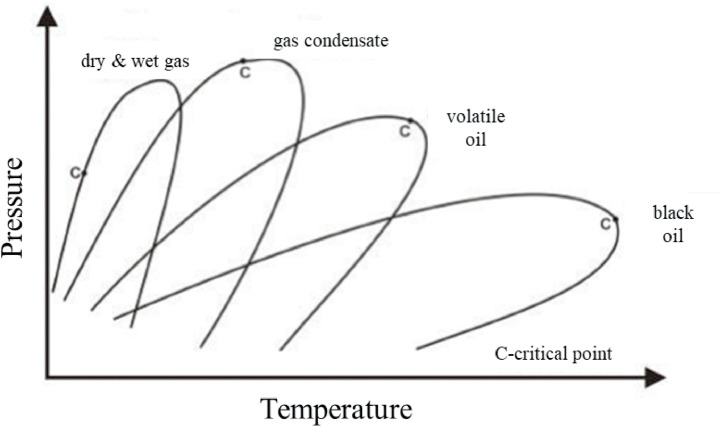
**Phase diagrams of the five petroleum reservoir fluids [**37**].**

**Fig. 4:**
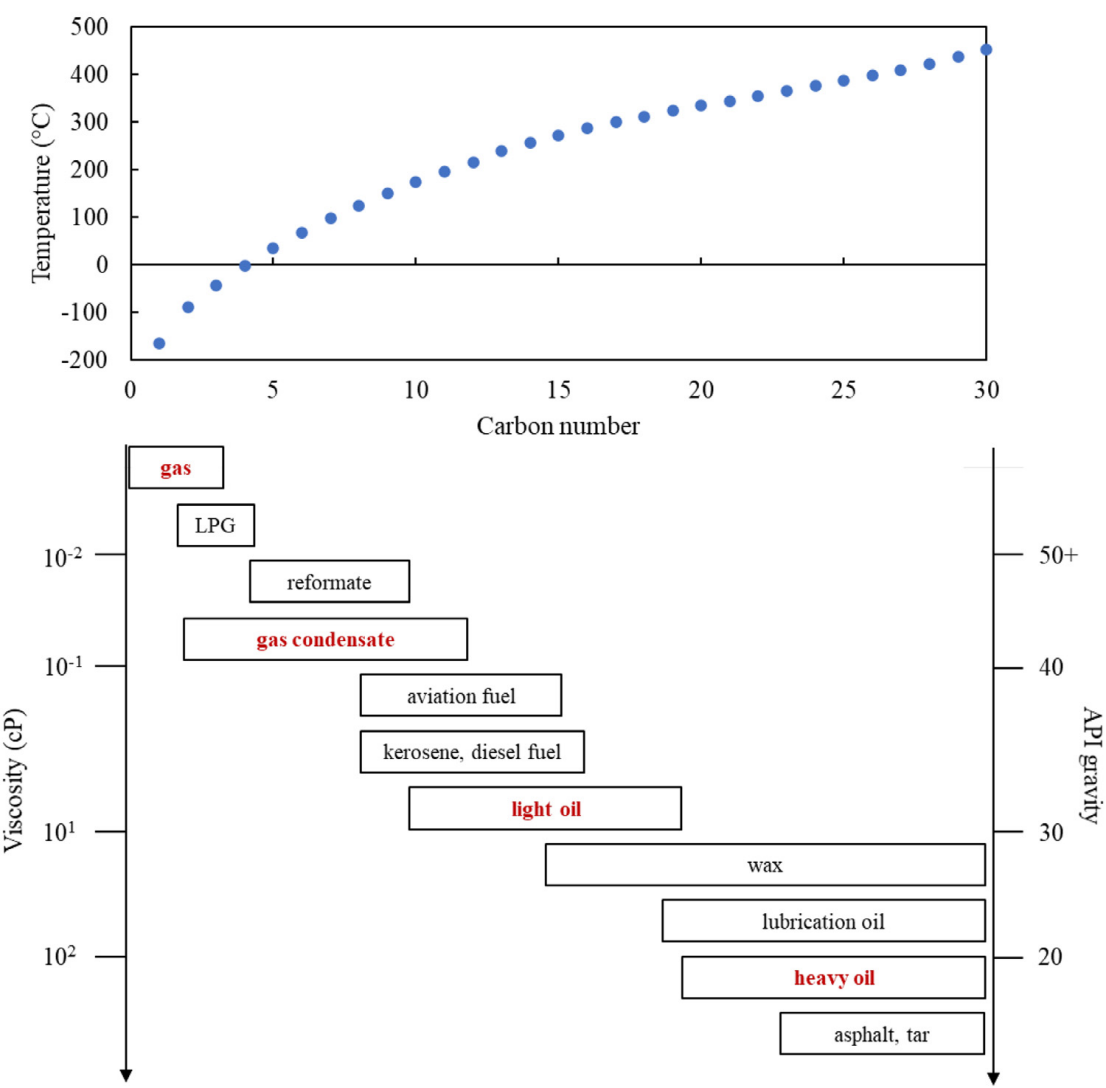
**Physical properties of hydrocarbons and petroleum products [**37**,**^38^**,**^41^**].**

This study emphasizes the origin of fossil fluids (i.e., petroleum reservoir fluids) without discussing derivative petroleum products (e.g., gasoline and diesel fuels). According to conventional petroleum classification, petroleum fluids can be divided into five major categories based on their physical and chemical properties, in increasing order of chemical complexity: dry gas, wet gas, gas condensate, volatile oil, and black oil [37]. Fig. 3 shows the phase diagrams of the five petroleum reservoir fluids and their general compositions and properties. Notably, the five petroleum fluids are different in terms of their phase diagrams, such as their single/two-phase area and critical point. The specific properties of the five petroleum fluids are listed in Table 1. As previously stated, current hydrogen-production technologies prefer low carbon and sulfur contents, but high-methane gaseous raw materials as feedstocks. Thus, dry/wet gases and gas condensates are better than the volatile and black oils currently used for hydrogen production. The five fossil fluids are presented together with their petroleum products in Fig. 4, which are plotted against the carbon number, viscosity, and API gravity. With an increasing number of carbons from a gas to gas condensate and light/heavy oils, the viscosity and API gravity also increase. Considering hydrocarbon reforming or pyrolysis reactions, gas and gas condensates are better feedstocks owing to the weak chemical bonding amongst low-carbon hydrocarbons [40]. Hence, dry/wet gas and gas condensates are better fossil fluids than light/heavy oils for hydrogen production from the perspective of fluid properties.

**Table 1:** **Physical properties of the five petroleum fluids [**38**]**.

Composition (mol.%)					
Component	Dry gas	Wet gas	Gas condensate	Volatile oil	Black oil
CO_2_	∼ 0.1	1–2	2–4	< 1	< 0.1
S	< 0.01	< 0.01	< 0.01	0.01–2	0.1–6
N_2_	1–2	0.1–0.5	< 1	< 1	< 1
C_1_	> 80	> 80	60–80	40–60	20–40
C_2–7_	5–15	< 10	10–20	10–20	∼ 10
C_7+_	–	< 1	< 12.5	12.5–30	> 30

Properties					

MW_C7+_	–	< 150	150–200	200–250	> 250
*γ*_C7+_	–	< 0.8	0.8–0.85	0.85–0.9	> 0.9
GOR (scf/STB)	∞	> 50,000	3300–50,000	2000–3000	< 2000
*γ*_API_	–	> 60	40–60	> 40	< 45
*P*_sat_ (psi)	–	3000–4000	> 6000	4000–6000	< 3000
*B*_sat_ (bbl/STB)	–	< 0.01	< 0.01	> 2	< 2
*ρ*_sat_ (lbm/ft^3^)	–	< 10	< 30	> 30	> 40
Colour	–	water-white	lightly coloured	brown or orange	dark or brown

In addition to physical properties, the resource availability and recovery technology of fossil fluids should be considered. Fig. 4 shows that fossil reservoir fluids are divided into three categories: natural gas, light oil, and heavy oil.

#### 2.1.1 Natural gas

**Fig. 5:**
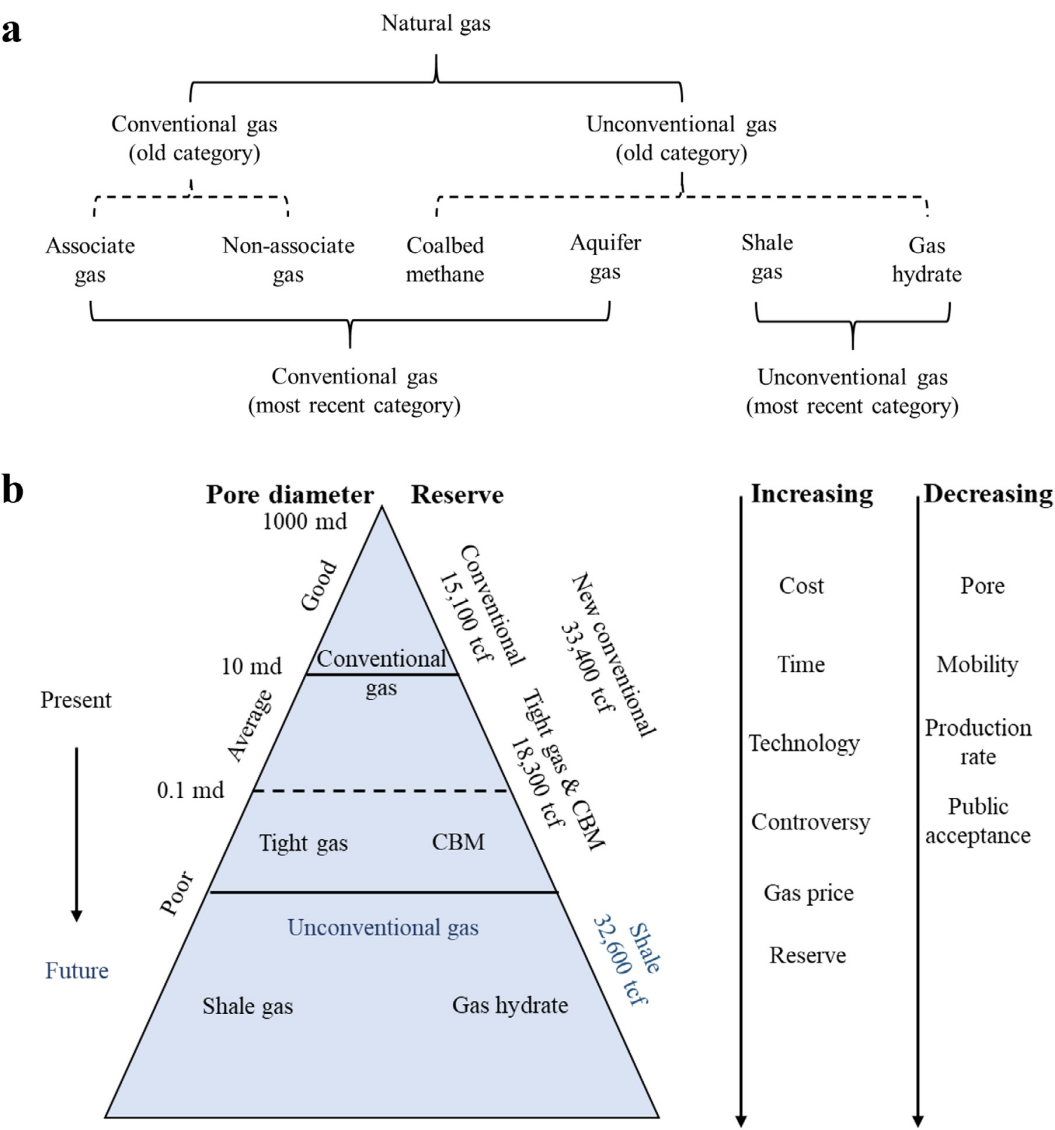
**(a) Categories of conventional and unconventional natural gas [**47**,**^48^**]. (b) Global natural gas pyramids [**49**].**

Natural gas is a colourless, shapeless, odourless combustible gas whose global reserves, according to the BP Statistical Review of World Energy (2020), were 198.8 trillion cubic meters as of the end of 2019. As shown in Fig. 5a, natural gas can be classified into conventional and unconventional categories based on its origin. Conventional gases are typically produced in hydrocarbon reservoirs, which are either gas-dominated with little or no oil (non-associated gas) or mixed with crude oil (associated gas). Non-associated gases originate from a geological formation with rich methane content but few high-boiling (compared with methane) hydrocarbons. Associated gases coexist with oil in the form of a solution gas or gas cap. Compared to non-associated gases, associated gases are richer in high-molecular-weight paraffinic components but sparser in methane [42]. The major subcategories of unconventional gases include shale gas, coalbed methane, gas hydrates, and aquifer gas. Coalbed methane is generated from coal seams and formed during the coalification process. Similar to that for shale gas, the primary component of coalbed methane is methane, with minor amounts of ethane, carbon dioxide, nitrogen, and sulfur compounds [43]. Aquifer gas denotes the dissolved gas in deep reservoir aquifers, whose composition is dependant on the specific pressure, temperature, and salinity conditions owing to the low solubility of methane in water [44]. Although aquifer gas is individually listed as a subcategory of unconventional gases, its usage in hydrogen production could be negligible owing to its unstable reserves. Shale gas is an emerging fossil gas with technological developments in its production (e.g., hydraulic fracturing) that has attracted attention owing to its wide distribution and abundancy. Shale gas refers to dry methane gas without oil and has been found in various geological formations, such as low-permeability shale, tight sandstone, carbonates, dolomite, limestones, and chalk [45]. Gas hydrates are another emerging gas resource formed via nucleation and crystal growth from the nuclei. Any gas can be hydrated at an appropriate temperature and pressure; however, natural gas hydrates, which primarily comprise methane and are known as combustible ice, are only formed under low temperature and high pressure conditions. Natural gas hydrates are commonly found in deep-sea or lake sediments and in terrestrial deposits in polar regions. In addition to temperature and pressure, several other factors influence the formation of natural gas hydrates, such as gas composition, water salinity, and the nature of the porous media [46].

A global natural gas pyramid, which includes both conventional and unconventional gases, is presented in Fig. 5b according to its specific characteristics. The total natural gas reserves are high, with 32,600 trillion cubic feet estimated to be in shale reservoirs (equal to the sum of all other gas reserves) [50]. Conventional gas resources and production have been previously discussed [51] and are not discussed here. Recently, an increasing number of energy statistics reports have categorized coalbed methane and aquifer gas as conventional natural gases, whereas unconventional gas primarily refers to shale gas and gas hydrates [52]. The IEA estimated that the remaining recoverable unconventional gas resources were equivalent to approximately 125 years of global natural gas consumption in the current mode, and on par with those of the remaining recoverable conventional gas reserves [45]. Although estimated reserves of gas hydrates could be 2–10 times greater than that of the currently known conventional gas reserves, the most relevant discovery and production technologies remain in the experimental phase. Therefore, no current technology can enable gas hydrate production in practice, and shale gas is a more feasible feedstock for hydrogen production owing to its large reserves and developed technology.

Shale gas resources refer to a gas-bearing shale system, which continuously accumulates natural gases as defined from multiple perspectives in the literature [53]. After reviewing all definitions, shale gas has been defined as a biogenic, thermogenic, or multi-genic gas that is continuously distributed in large-scale organic-rich shale systems with nanoscale pores, multi-lithology traps, and short migration distances [[54], [55], [56]]. As demonstrated in Fig. 5b, with decreasing pore size, fluid mobility, and production rate, the successful exploitation of unconventional gas resources would require higher production and operation costs, longer life cycles, higher requirements of technology and market prices, and more societal controversy. Based on previous studies, shale gas reservoirs have the following five characteristics:

(1) Large-scale continuous distribution. Shale is not only a source rock but also a reservoir with abundant gases and storage ability. Its accumulation in authigenic reservoirs results in the continuous distribution of shale reservoirs on large-scale slopes and in depression areas.

(2) Widespread lamina and bedding development. Lamina and bedding facilitate the connection of inorganic mineral and nanoscale organic pores and the formation of a high-speed channel for horizontal fluid migration in the shale reservoir. This feature significantly improves the horizontal fluid flow capability and provides an opportunity for the horizontal well to enhance fluid production.

(3) Abundant nanoscale organic pores. Gas-bearing shale is tightly compacted such that nanoscale pores are the primary spaces for gas accumulation. Nanoscale pores account for over 90% of the total pores in shale, with pore diameters ranging from several to hundreds of nanometers and permeabilities on the order of 10^−9^ to 10^−6^ µm.

(4) Thermogenic-gas-dominated composition. Thermogenic gas is prevalent in shale gas resources in dissociated (free gas) and adsorbed (adsorbed gas) states.

(5) High requirements for exploitation technologies. The combination of a horizontal well and fracturing (mostly hydraulic fracturing) is the most widely applied technology that is effective in ensuring high shale gas production, but is also controversial due to its side effects.

Although the reserves of unconventional gas resources (primarily shale gas) are almost equal to those of conventional gas, the challenges associated with the development of unconventional resources are significant. Based on the five listed characteristics of unconventional gas resources, three major challenges and two uncertainties are summarized: challenges in geological exploration, technology and viability development, and regulatory and public acceptance, as well as uncertainties in operator/service sector capacity and natural gas pricing. To date, the first two challenges have been addressed by the U.S. oil and gas industry, whereas the other challenges and uncertainties require further investigation [45].

#### 2.1.2 Crude oil

**Fig. 6:**
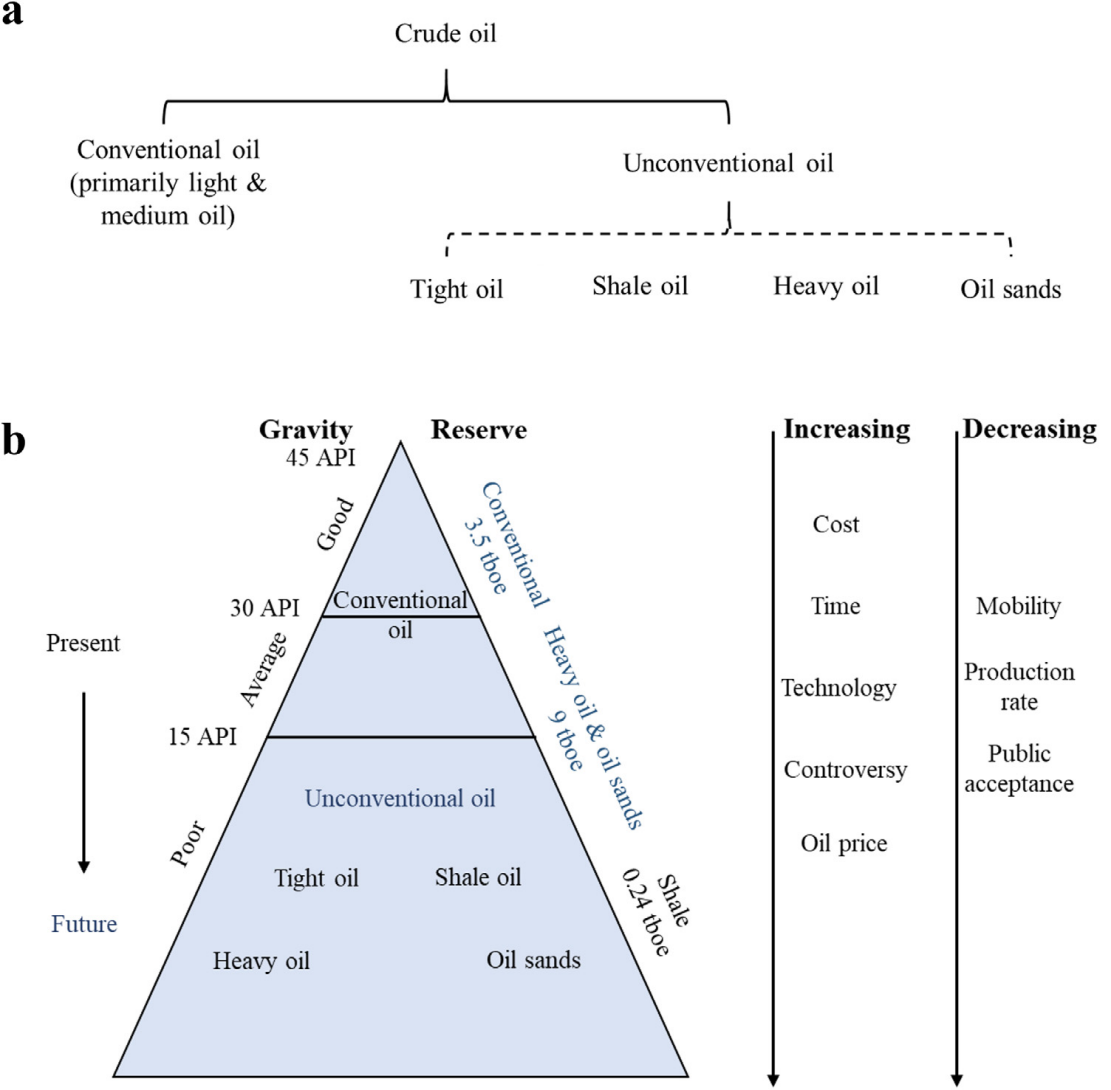
**(a) Categories of conventional and unconventional crude oil [**58**]. (b) Global crude oil pyramids [**58**].**

Crude oil is a naturally occurring, yellowish-black liquid in subsurface geological formations. It is typically categorized as a conventional or unconventional oil based on reservoir characteristics and fluid physical properties, as shown in Fig. 6. Conventional oils primarily include light (API > 31.1°) and medium (22.3° < API < 31.1°) oils, whereas unconventional oils include tight, shale, and heavy oils and oil sands [37]. Similar to natural gas, unconventional oil resources increase facility and operation costs, production time, technology requirements, and oil prices while decreasing fluid mobility, production rate, and public acceptance. Hence, conventional oil remains a potential primary resource as the feedstock for hydrogen production because of its oil-in-place and techno-economic advantages over unconventional oils. Numerous studies have been conducted to investigate and review conventional oil resources. These details can be found in the literature [[47], [51], [57]] and are not discussed here. Regarding unconventional oil resources, the estimated heavy oil reserves are higher than those of tight and shale oils. Considering that shale oil shares similar compositional properties and technical factors with shale gas, only heavy oil is briefly introduced in the following paragraphs.

**Fig. 7:**
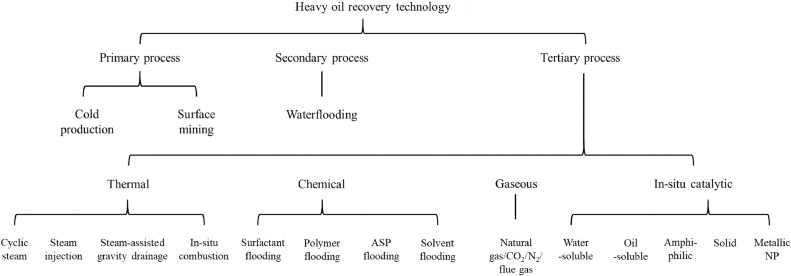
**Summary of heavy oil recovery technologies [**60**].**

Heavy oil is called “heavy” because its density or specific gravity is higher than that of conventional oils. For example, the API gravity of heavy oil is < 22.3°. In addition, compared to conventional light and medium oils, heavy oil is more complex owing to its inherent properties of high viscosity, high carbon–hydrogen ratio, and high heteroatom content. The differences between heavy and conventional oils are attributed to differences in their fluid compositions. Heavy oil components are high-molecular-weight hydrocarbons, have carbon atom counts of ˃ 60 and high boiling points, and are generally categorized as saturated, aromatic, resin, and asphaltene (SARA) according to their polarity and polarizability [59]. The SARA analysis is a typical method for quick compositional evaluation, and its details can be found in the literature. In addition to common components, such as linear, branched, and cyclic saturated hydrocarbons, aromatic rings, resin, and asphaltene, SARA contains certain amounts of nitrogen, oxygen, sulfur, and metal compounds. Although the specific SARA proportions could differ due to geological differences, most heavy oils typically contain high contents of asphaltene and sulfur compounds [60]. Owing to the unique properties of heavy oil, the recovery technologies that have been applied in the petroleum industry are discussed. To the best of our knowledge, all common heavy oil recovery technologies are summarized in Fig. 7. Although they show relatively high efficiency and low risk, cold production and surface mining are the primary and most initially applied processes and are limited by the geological characteristics of reservoirs, such as depth and structure [61]. The secondary recovery process comprises water flooding, including modified low-salinity brines, that are widely applied in light and medium oil recovery but are not applicable for heavy oils owing to the large viscosity difference between water and heavy oil, which induces low sweep efficiency [62].

Considering the limitations of primary and secondary processes, various tertiary processes have been developed and applied to increase heavy oil recovery. As shown in Fig. 7, the tertiary recovery process can be divided into four major categories based on process features: thermal, chemical, gaseous, and catalytic. The specific advantages and disadvantages of each process have been previously described [60]. Briefly, thermal processes typically require an intensive energy supply and generate numerous greenhouse gases, which increase environmental and economic costs. Instead of reducing oil viscosity using thermal methods, chemical-based approaches improve the mobility of viscous oils via IFT reduction, mobility control, and wettability alteration with solvent addition (e.g., surfactants and polymers). The key limitations of chemical methods are their high capital costs, complex operational processes, and controversial environmental impacts. Gas injection is typically not effective for heavy oil production owing to viscous fingering and mobility override. The following three methods can be adopted to mediate the gas limitations: 1) Attain a supercritical state in a reservoir with a depth of > 800 m; 2) utilize a synergistic combination of chemical and gas solvents in the foam state; and 3) inject alternately into the water.

### 2.2 Fossil mineral

**Fig. 8:**
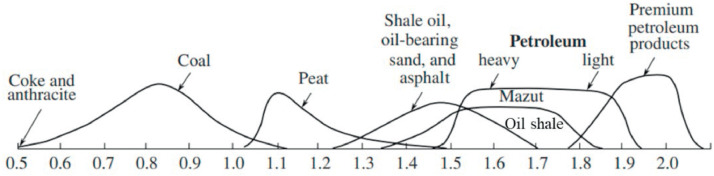
**H/C aromatic ratios of different fossil fluids and minerals [**63**].**

Coal is an important fossil mineral that can be used for dehydrogenation. However, as demonstrated in Fig. 8, the H/C atomic ratio of coal is low (0.4–1.0), and excessive carbon emissions are typically generated in coal processing and conversion [63]. Thus, the emphasis of this review was on oil shale, which is another important fossil mineral that can potentially be converted into clean products. The most significant advantages of oil shale over other fossil minerals are its high H/C atomic ratio of approximately 1.7 and its unique organic matter composition, which enables the production of a wide range of petroleum products, such as premium lubricating oils or motor fuels. Another important advantage is that global resources comprise approximately 2.9 billion barrels of oil shale. Oil shale can be converted into fuel and chemical products via two methods: 1) The organic matter of shale can be converted to a mixture of hydrogen and carbon oxides; and 2) oil shale can be pyrolyzed to form gaseous, liquid, and solid products. Although the second method has been practiced, the first method lacks an in-depth understanding and requires further study. Oil shale pyrolysis refers to the thermal degradation of solid raw materials and the production of olefins and aromatic compounds. Instead of pyrolysis, dry distillation, low-temperature carbonization, and coking are often used for solids in slightly different processes. The pyrolysis of oil shale is affected by the following factors: origin and type of organic matter, processing temperature and pressure, heating source and rate, residence time in the reactor, and grain size composition. In contrast to fossil fluids, oil shale conversion requires complex chemical processing owing to the presence of a mixture of kerogen, organic matter, and fossil fluids. Moreover, the converted products of oil shale contain high contents of O-, S-, and N-organic compounds and a low hydrogen percentage; therefore, they probably need purification before being applied as fuels.

## 3 Hydrogen technology

Most fossil-fuel-sourced hydrogen is produced by hydrocarbon reforming and pyrolysis [[64], [65], [66], [67]], and renewable sources typically include biomass and water processes [[68], [69], [70]]. In this section, hydrocarbon reforming processes, including steam, plasma, and aqueous-phase reforming, POX, ATR, and hydrocarbon pyrolysis, are reviewed from a technical perspective.

### 3.1 Hydrocarbon reforming

Hydrocarbon reforming is well developed in various formats and refers to reforming technologies that convert hydrocarbon fuels to hydrogen [71]. Steam, which initiates endothermic reactions such as steam reforming, or oxygen, which enables exothermic reactions such as POX, or the combination of steam and oxygen for autothermal reactions, are required reactants (e.g., nanoparticles) in addition to feedstock hydrocarbons [72]. Most hydrocarbons are produced with sulfur or sulfide compounds, which negatively affect the reforming catalyst and entire conversion process [73], thereby negatively affecting the environment [74,75]. In addition to the regular reforming and cleanup setup, accessories such as compressors, heat exchangers, coolers, pumps, and desulfurization units are necessary for the reforming plant.

#### 3.1.1 Steam reforming

The steam reforming technique involves the conversion of hydrocarbons and steam to hydrogen and carbon oxides (e.g., mono- and dioxide). Steam reforming processes include feedstock reforming, synthesis gas generation, the water–gas shift (WGS) reaction, gas purification, and possibly carbon removal [76]. The feedstock materials could be methane, natural gas, methane-containing light hydrocarbon mixtures, or naphtha, but preferably no sulfur compounds. After feeding the materials mentioned above, the reforming reactions are typically operated at high temperatures (850–900 °C), moderate pressures (up to 3.5 MPa), and at a fair steam–carbon ratio (commonly 3.5) to prevent coking on the catalyst surface and produce high-purity hydrogen [77]. The primary chemical reactions in the reforming process are as follows:

(1) $$C_{m}H_{n}+mH_{2}O\to m\text{CO}+\left(m+\frac{1}{2}n\right)H_{2}$$

For example, in SMR, *m* and *n* are equal to 1 and 4, respectively. The products (primarily hydrogen, carbon monoxide, and carbon dioxide) of the reforming reactions are processed through the WGS reactor, and heat can be recovered and refed into the reforming reactor to produce more hydrogen from the carbon monoxide-steam reactions.

(2) $$\text{CO}+H_{2}O\to CO_{2}+H_{2}$$

Subsequently, the mixtures must pass through the process of methanation and/or CO_2_ removal before purification to produce hydrogen. This occurs through methods such as pressure swing adsorption, membranes, or other methods. The chemical reaction for methanation is as follows:

(3) $$\text{CO}+3H_{2}\to CH_{4}+H_{2}O$$

**Fig. 9:**
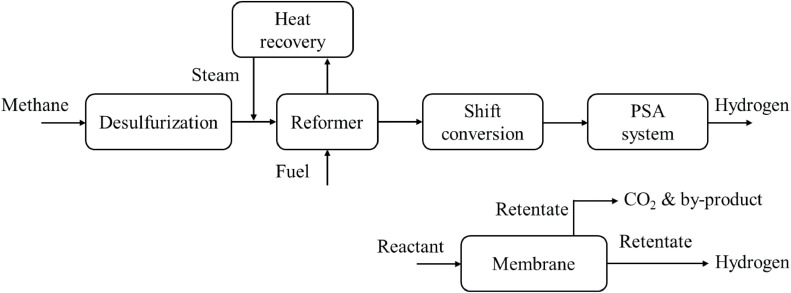
**Steam methane reforming with and without the membrane flowchart [**71**].**

The entire steam reforming process is illustrated as a simplified flowchart in Fig. 9.

With increasing production efficiency requirements and concerns regarding global climate change, promising membrane reactors and carbon capture and storage (CCS) technologies are embedded in conventional steam reforming processes. As a major technology for industrial-scale hydrogen production, steam reforming has been investigated by integrating selective membranes either in reforming reactors or downstream of the entire reforming reaction process [78]. Fig. 9 shows a flowchart of the membrane-integrated steam reforming process, from which the conventional reforming method could benefit by means of inorganic-based membrane reactors [79]. Membrane-integrated reactors, using Pb-based materials as examples, enable a higher conversion efficiency (up to 90–95%) compared with traditional SMR (70–85%) at approximately 400 °C lower temperatures [80]. In addition, as demonstrated in Fig. 9, the ability of the steam reforming process to be coupled with CCS technology strongly reduces CO_2_ emissions and facilitates transitions to sustainable green production.

#### 3.1.2 Partial oxidation

POX refers to the conversion of hydrogen and carbon oxides from steam, oxygen, and hydrocarbons. Its major processes are similar to those of steam reforming. POX can be processed with and without catalysts at approximately 950 and 1150–1300 °C, respectively. The feedstock of the catalyst process covers methane to naphtha, whereas for the non-catalytic process, only hydrocarbons (including light and heavy oils and coal) can be used as feed materials [77]. Desulfurization is necessary to prevent the formation of poisonous sulfur compounds before inputting the feedstock into the reformer. Although the catalyst could increase process efficiency, its pricing also needs to be considered [[81], [82], [83]]. Subsequently, pure oxygen and steam are required to partially oxidize the hydrocarbon feedstock. The primary chemical reactions of the catalytic and non-catalytic processes in the reformer are shown in Eqs. (4) and (5), respectively.

Catalytic:

(4) $$C_{m}H_{n}+\frac{1}{2}mO_{2}\to m\text{CO}+\frac{1}{2}nH_{2}$$

Non-catalytic:

(5) $$C_{m}H_{n}+mH_{2}O\to m\text{CO}+\left(m+\frac{1}{2}n\right)H_{2}$$

A subsequent WGS reaction can be implemented (Eq. (2)), and the produced carbon monoxide can be oxidized as follows:

(6) $$\text{CO}+O_{2}\to CO_{2}$$

**Fig. 10:**
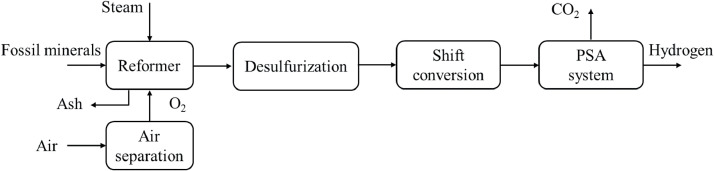
**Partial oxidation process flowchart [**71**].**

Before purifying and producing hydrogen as the final product, further processing of the syngas (e.g., methanation or CO_2_ removal) can be implemented to save energy. A flowchart of the POX process is shown in Fig. 10.

Compared to the SMR method, POX has a higher capital expense owing to the oxygen plant and cost of intensive desulfurization processes. However, POX is the most appropriate method for hydrogen production when the feedstocks are heavy hydrocarbons (e.g., heavy oils) and coal (known as coal gasification) [28]. For coal gasification, *m* and *n* in Eq. (5) are equal to 1 and 0, respectively. As shown in Fig. 4, coal gasification comprises a series of steps that are similar to that of POX, except for additional costs by processing the relatively un-reactive fuel as a solid and excess ash removal. Considering the lower hydrogen-to-carbon ratios of the heavy feedstock in POX (compared to other methods such as SMR), large quantities of hydrogen come from the steam, particularly for heavy hydrocarbons and coal gasification. Specifically, steam water supplies approximately 69% and 83% of the hydrogen for heavy oil and coal processing, respectively [77]. Regarding the conversion efficiency and capital/operational costs, feeding heavy hydrocarbons is preferred over coal.

#### 3.1.3 Autothermal reforming

**Fig. 11:**
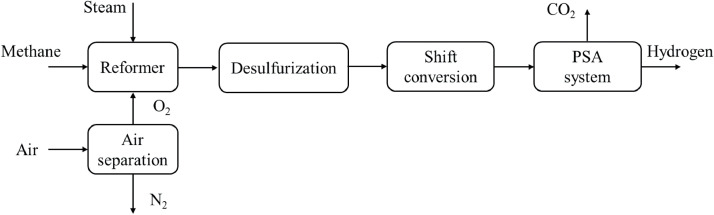
**Autothermal reforming process flowchart [**84**].**

ATR is a combination of steam reforming and POX that utilizes exothermic POX as a heat source and endothermic steam reforming to improve hydrogen production [28]. A flowchart of the ATR process is shown in Fig. 11. Oxygen and steam are co-injected into the reformer to simultaneously initiate the oxidation and reforming reactions via the following chemical reaction:

(7) $$C_{m}H_{n}+\frac{1}{2}mH_{2}O+\frac{1}{4}mO_{2}\to m\text{CO}+\left(\frac{1}{2}m+\frac{1}{2}n\right)H_{2}$$

This optimized operational scheme, which uses methane as a feedstock, has an inlet temperature of approximately 700 °C and steam–carbon and oxygen–carbon ratios of 1.5 and 0.45, respectively. Using the optimum conversion scheme, the maximum hydrogen production was 2.8 times that of the normal process [[85], [86]]. ATR methods include desulfurization, shift conversion (WGS), and hydrogen purification, which are conducted in steam reforming and ATR processes. Similar to the steam reforming process, the traditional ATR method has been modified to improve hydrogen conversion efficiency by integrating a membrane. For example, certain studies have found that using a Pd membrane coupled with an ATR reactor resulted in up to a 20% reduction in the fuel processor volume and a slight increase in system efficiency [84]. Moreover, higher methane conversion, lower hydrogen and carbon dioxide retentate sides, and higher carbon monoxide concentrations were observed [87]. Although the overall system efficiency could be improved by integrating a membrane, this modification faces certain limitations, such as membrane degradation owing to the high temperature requirement (approximately 900 °C) and the complex design of the ATR reactor with the membrane [88].

Amongst the three processes in Sections 3.1.1–3.1.3, the POX and ATR methods require expensive and complex oxygen separation units to feed pure oxygen into the reactor, and the product gas is diluted with nitrogen. All three processes produce abundant carbon monoxide, and one or more WGS reactors, which are typically high- or low-temperature reactors, are employed. The high-temperature (> 350 °C) reactor exhibits rapid kinetics, which is restricted by the limited amount of carbon monoxide that is shifted by thermodynamics. Thus, a low-temperature reactor (210–330 °C) is required to convert a larger fraction of carbon monoxide with low efficiency. High-temperature WGS reactors commonly use iron catalysts, whereas low-temperature reactors often use copper catalysts.

#### 3.1.4 Plasma reforming

Though it shares similar reforming reaction principles with that of conventional reforming, plasma reforming is different because the energy supplied for the reforming processes is typically generated with electricity or heat [28]. With the injection of steam or water, *H*^+^, OH^−^, and O^2−^ radicals are formed and participate in oxidative and reductive reactions. Generally, plasma reforming can be categorized into thermal and non-thermal processes.

**Fig. 12:**
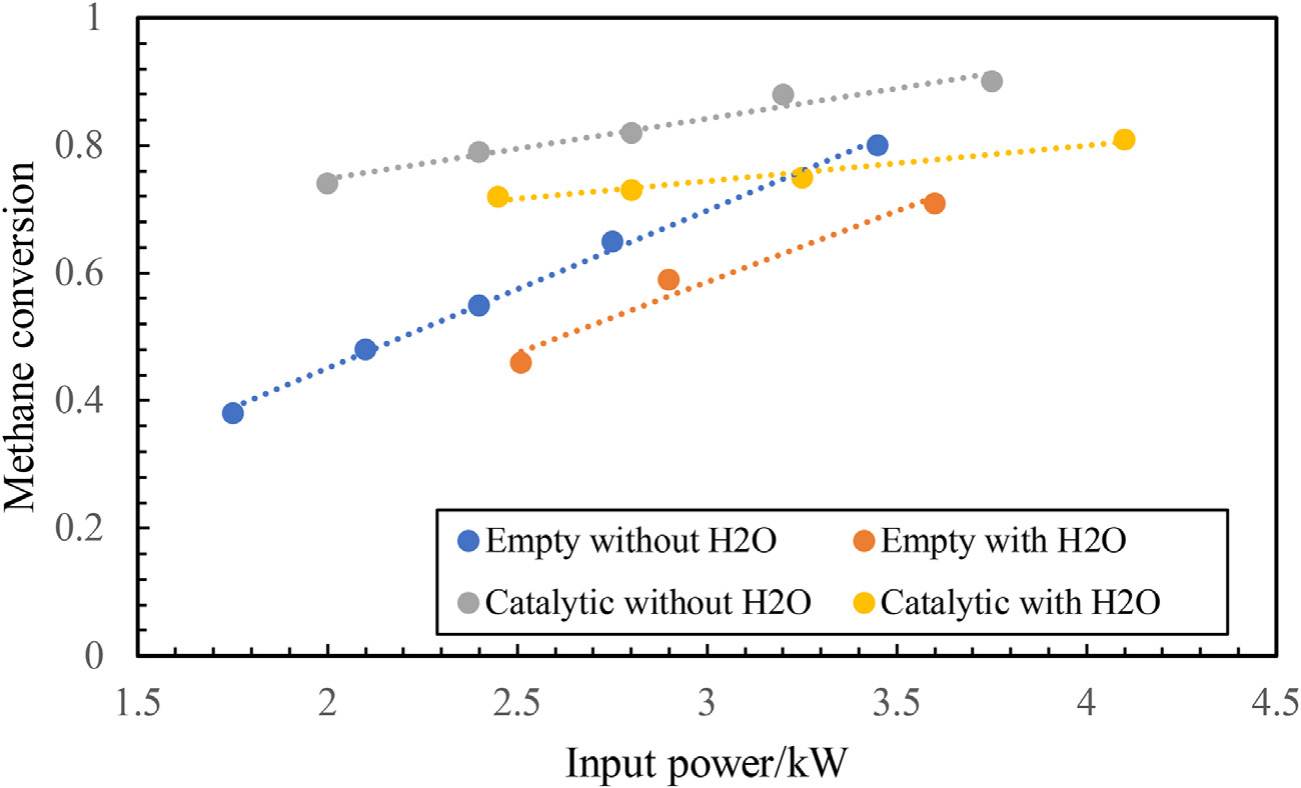
**Required input power for methane conversion via plasma reforming [**88**].**

Thermal plasma reforming typically has a high efficiency and requires a high electric discharge (> 1 kW) [88]. Abundant power supplies are required to raise the temperatures of electrons and bulk species up to extremely high temperature of 4726.85–9726.85 °C and cool down the vaporizing process post-reforming [89,90]. As a specific example, Fig. 12 shows the power supply for methane conversion and hydrogen production via thermal plasma reforming. The figure shows that producing 1 kg of hydrogen consumes approximately 16 MJ of energy [88]. Hence, reducing the consumption of power remains a challenge for thermal plasma reforming.

Non-thermal plasma reforming only requires high temperatures for the electrons, and the majority of power for exciting the bulk species can be conserved [[89], [91], [92], [93], [94]], which is favored with more diverse formats of reformers. Four different types of reformers have been discussed in the literature for non-thermal plasma reforming: corona discharge, gliding arc plasma, dielectric barrier discharge (DBD), and microwave plasma. The primary difference between the four types is the specific method used to control the current and discharge. Particularly, corona discharge uses a static discharge to generate plasma, whereas the others create plasma with a dynamic discharge [89]. The corona discharge and gliding arc plasma reformers share a similar design setup but operate in different modes, with the reactor comprising two electrodes. In corona discharge, plasma is generated between the electrodes through the length, whereas plasma is created by pushing the gas down to the end of the reactor in the gliding arc plasma process [95,96]. The plasma in the corona discharge can be quickly generated using fast-rising electric pulses (i.e., 10 ns rise time plus 200 ns pulse), but in the gliding arc plasma process, the arc needs to be turned off when the gas reaches the end of the reactor and before another round of gas injection. Thus, corona discharge outperforms power-saving and processing efficiency. However, the gliding arc plasma process is more user-friendly, with flexible operation processing and the use of a simple power supply. The DBD reactor is annularly configured with a millimetre gap between the nonconductive material-encased voltage electrodes [97]. Co-feeds of methane, water, carbon monoxide, and dioxide are used to create hydrogen and hydrocarbons. This method is of special interest because greenhouse gases can be eliminated during the process in addition to hydrogen and by-product hydrocarbon production. In contrast to these three plasma processes, microwave plasma uses microwaves rather than electric arcs to generate hydrogen. However, with current technology, evidence indicates that this process could require more energy than the generated hydrogen, which is produced using fuel cells.

#### 3.1.5 Aqueous phase reforming

Aqueous-phase reforming (APR) is a relatively new process for producing oxygenated carbohydrates or hydrocarbon-processed hydrogen [[98], [99], [100]]. Previous studies have reported that APR reactors are typically operated at temperatures of 220–270 °C and pressures up to 25–30 MPa, and most research has focused on Pt- and Ni-based catalysts due to their high activity and low cost, respectively. The reforming reactions are complex, and the pathways for reforming and WGS follow Eqs. (1) and (2), respectively. Although the APR process is under development, its current advantages can be summarized as follows: 1) process simplification by eliminating feedstock vaporization and degradation as well as multiple reaction steps; and 2) process efficiency improvements by selecting proper species of feedstock and catalyst [101]. However, APR is limited by catalyst instability in long-term tests and large-space requirements in practical applications [102]. Hence, improving the catalyst activity and durability while maintaining an acceptable setup space is critical for future APR development.

### 3.2 Hydrocarbon pyrolysis

Hydrocarbon pyrolysis is a process that requires hydrocarbons as the only feedstock, thereby avoiding steam or oxygen. They are thermally decomposed into carbon and hydrogen via the following chemical reactions [86]:

(8) $$C_{m}H_{n}\to mC+\frac{1}{2}nH_{2}$$

Specific processes can differ according to the feed hydrocarbons. For light liquid hydrocarbons with boiling points in the range of 50–200 °C, thermocatalytic decomposition directly produces carbon and hydrogen, as demonstrated in Eq. (8) [103]. However, when the feedstocks have heavy fractions with boiling points >350 °C, hydrogen is generated via the following steps (i.e., hydrogasification and methane cracking):

(9a) $$CH_{1.6}+1.2H_{2}\to CH_{4}$$

(9b) $$CH_{4}\to C+2H_{2}$$

(9c) $$CH_{1.6}\to C+0.8H_{2}$$

**Fig. 13:**
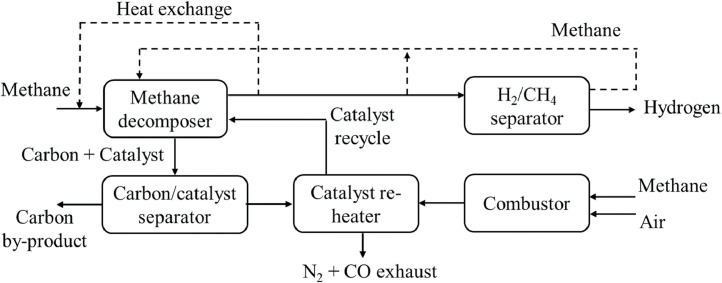
**Methane pyrolysis process flowchart [**71**].**

In principle, neither steam nor oxygen is involved in these chemical reactions. Thus, no carbon oxides (e.g., carbon monoxide and carbon dioxide) are formed, and no secondary processing (e.g., WGS or CO_2_ removal) is required. A simplified flowchart of the hydrocarbon pyrolysis with methane is shown in Fig. 13. However, when steam or air is accidentally involved under certain conditions, substantial carbon oxides can be produced, which may cause negative environmental impacts. Hence, it is critically important to control the moisture content of feedstocks to avoid the generation of greenhouse gases. Overall, hydrocarbon pyrolysis is advantageous because of its low capital investment and energy requirements, simple operational design and processes, and clean, carbon-free byproducts [104,105]. Notably, WGS or CO_2_ removal steps are not required in the pyrolysis processes, and capital investments for commercial-scale plants are substantially (approximately 25–30%) lower than those for steam reforming or POX. From an environmental perspective, pyrolysis is favored because hydrogen is produced from hydrocarbon decomposition. Conversely, large amounts of carbon oxides are produced from steam reforming and POX, which require CO_2_ mitigation techniques, such as CCS [105]. Although hydrocarbon pyrolysis performs well, it could be limited by the form of carbon, which probably requires special facility design and modification for future large-scale practical applications. Further improvements, such as coupling with functional membranes or carbon removal facilities, will increase hydrogen conversion efficiency in a cleaner manner and enable pyrolysis to play a more important role in the near future.

**Fig. 14:**
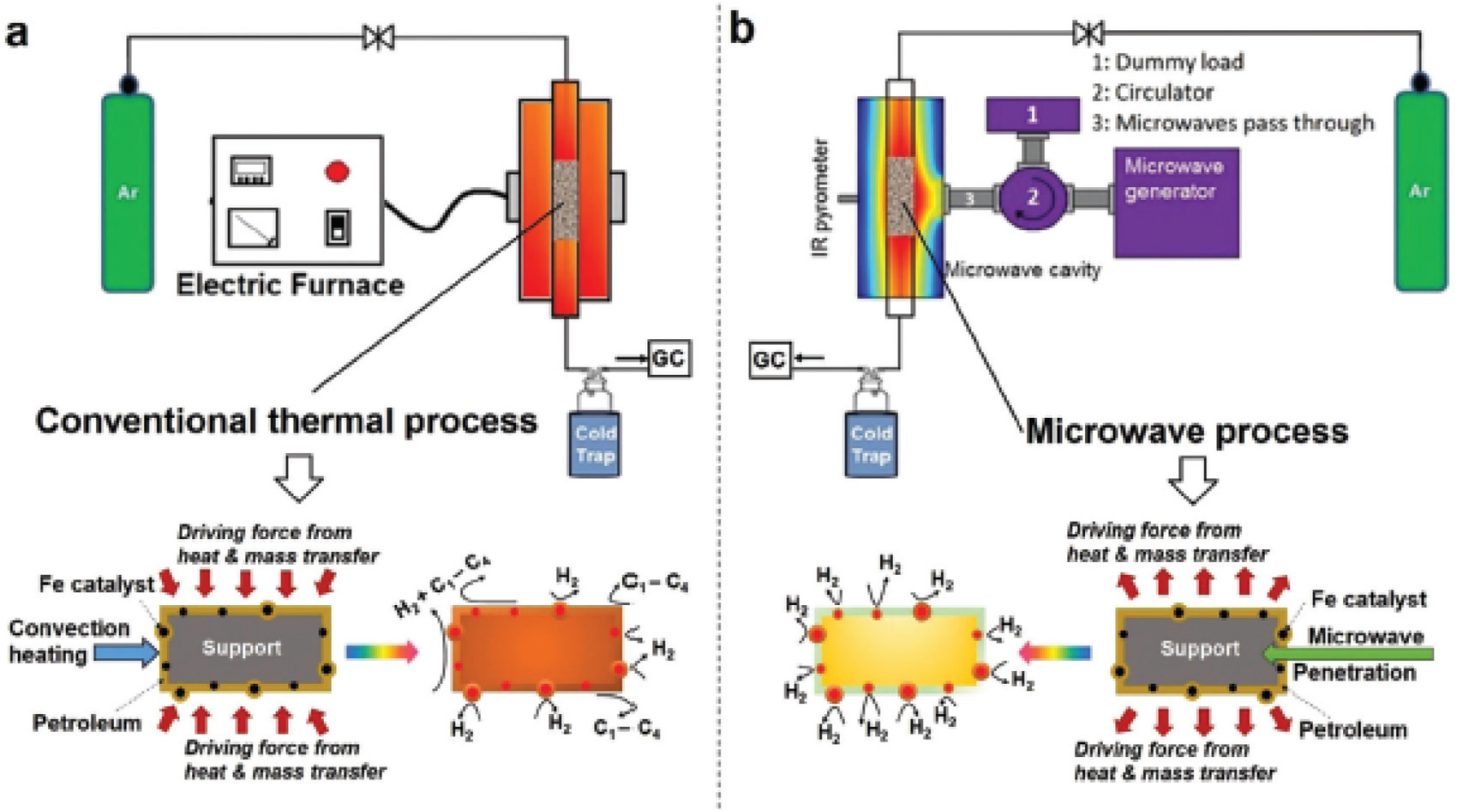
**Schematic diagrams of the (a) thermo- and (b) microwave-catalytic hydrocarbon decomposition processes [**27**].**

A new series of carbon-free hydrocarbon decomposition reactions (hydrocarbon decomposition here is differentiated from pyrolysis processes) with the assistance of catalysis is proposed. They are differentiated in terms of the activation sources, such as thermo- and microwave-catalytic processes [27,103]. Thermal energy affects catalyst particles via external heat radiation, convection, and conduction, whereas microwave energy is transferred directly through molecular interactions. Schematic diagrams of the thermo- and microwave-catalytic hydrocarbon decomposition processes are shown in Figs. 14a and b. Unlike heat transfer that gradually passes through the surrounding hydrocarbon fluids before reaching the catalyst, microwave irradiation directly interacts with the catalyst without heating the surrounding medium. This attribute not only causes rapid heating of the microwave-absorbing catalyst particles, but also facilitates potential product hydrogen selectivity. Consequently, this increases the overall reaction rate compared to conventional thermal heating. Moreover, in conventional thermal heating, the temperature of the hydrocarbon surroundings becomes higher than that of the catalyst with the development of the process, which causes the hydrocarbons to either decompose over the catalyst support or self-decompose, resulting in the production of various compounds. Thus, microwave-catalytic processes have the potential to be carbon-free, whereas conventional thermal processes can possibly produce carbon compounds in addition to hydrogen.

## 4 Resource & technology evaluations

The conventional hydrocarbon reforming and pyrolysis processes generate carbon emissions. For example, the SMR, POX, and ATR processes generally generate up to 0.25–0.33 m^3^ CO_2_/m^3^ hydrogen produced. Conversely, hydrocarbon decomposition has lower carbon emissions (approximately 0.05 m^3^ CO_2_/m^3^ hydrogen produced) or can be carbon-free if part of the produced hydrogen is used as fuel [106]. The overall theoretical energy requirement for each mole of hydrogen produced is approximately equal to 43.3 and 37.8 kJ in the processes of SMR and the conventional thermal decomposition of methane, respectively [25]. Moreover, hydrocarbon thermal decomposition is a simple one-step process without energy- and material-intensive gas separation or carbon removal stages. From a techno-economic perspective, hydrogen production from thermal decomposition costs $58/1000 m^3^ hydrogen, which is lower than that from SMR ($67/1000 m^3^ hydrogen) [25]. The capital cost component of thermal decomposition is 12.8%, whereas those of SMR and POX are 29.1% and 47.9%, respectively. Thus, the thermal decomposition of hydrocarbons outperforms the most common hydrocarbon reforming and pyrolysis processes owing to its energy efficiency, economic effectiveness, and environment-friendly features.

In this review, thermal and catalyst-assisted decomposition were compared and analysed. The most important reason for adding a catalyst is to mitigate extreme operational conditions (e.g., reduce the maximum temperature) and improve the reaction efficiency. amongst the different types of catalysts, transition metals demonstrate remarkably high activity, facilitating hydrocarbon decomposition reactions. However, there is no consensus on the selection of the most active metals for hydrocarbon decomposition [107,108]. An approximate general order of methane activation rates with transition metals can be obtained from the literature: Co, Ni, Rh, Ru > Pt, Ir, *Re* > Cu, Fe, Pd, Mo, and W [25,109]. Notably, metal activity is not the only factor to be considered in hydrocarbon decomposition. For example, although its activity is low, Fe is one of the most commonly used metal catalysts owing to its easy access and low cost. Notably, even the specific metal activity requires case-by-case analyses because it can be affected by the pressure, temperature, material of the catalytic support, and their induced hydrogen-metal interactions. Carbon-based catalysts have been used for hydrocarbon decomposition to address the limitations of metal catalysts and to improve their catalytic efficiency. Carbon-based catalysts perform better than metal catalysts by producing lower carbon emissions and sulfur-free compounds with high fuel flexibility, but with no catalyst regeneration. However, as mentioned in Section 2.2, the activation energy source is crucial for catalytic hydrocarbon decomposition, and using electromagnetic radiation, such as microwave activation energy, is better than using the conventional thermal method.

**Fig. 15:**
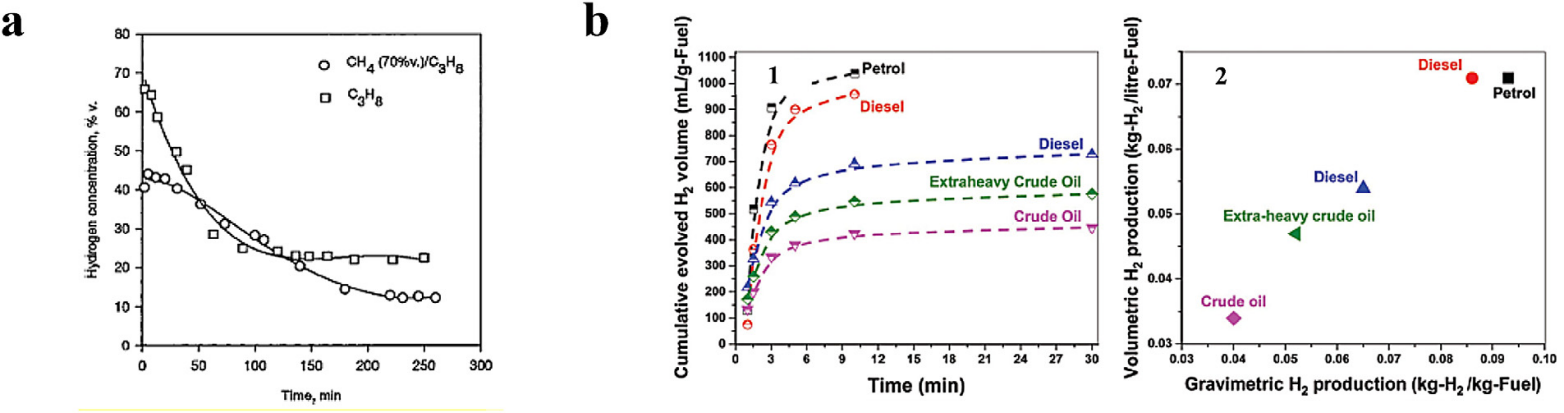
**(a) Conventional thermal decompositions of propane and a methane/propane mixture (70 vol.% methane) with activated alumina at 850 °C. (b) Microwave-assisted iron catalytic decomposition of fossil fuels: (b1) cumulative hydrogen volume and (b2) per volumetric and gravimetric fuel consumption-induced hydrogen productions [**27**].**

From a thermodynamic perspective, the decomposition (pyrolysis) of liquid hydrocarbons is more favourable than that of gaseous hydrocarbons because 1.5–2 times less energy is required to produce a unit volume of hydrogen. However, the amount of hydrogen obtained from liquid hydrocarbons is lower than that obtained from gaseous hydrocarbons. This is mostly caused by the heavy residual fractions of liquid hydrocarbons. The direct decomposition of such liquid hydrocarbons could generate coke because of the sulfur and metals present in the heavy residual fractions, resulting in less hydrogen being produced. As demonstrated in Fig. 15a, the carbon number of gaseous hydrocarbons does not affect the amount of hydrogen produced, whereas high-carbon hydrocarbons perform better for liquid hydrocarbons (shown in Fig. 15b) [27]. Thus, the suggested order of fossil fluids for hydrogen production is gaseous hydrocarbons > heavy liquid hydrocarbons > light liquid hydrocarbons.

Another important by-product of thermo/microwave-catalytic hydrocarbon decomposition is the carbide compounds. These could provide a potential solution to decarbonize the whole process (i.e., fossil fluid-feed hydrogen productions) and function as valuable feed for subsequent conversions. Previous studies have revealed that most carbon byproducts of efficient catalytic-assisted hydrocarbon decomposition reactions exist in the solid phase [110,111]. Compared with gaseous or liquid CO_2_ in CCS processes, solid elemental carbon is more suitable for capture and storage from a technical and ecological perspective. For example, 1 kg of diesel can be used to produce hydrogen using a microwave-catalytic decomposition process and concurrently generate 0.961 kg of elemental carbon as a coproduct. Moreover, the combustion of the same amount of diesel causes 3.155 kg of CO_2_ emissions (equivalent to 1.606 m^3^ to the atmosphere). The conversion and utilization of carbon by-products are attractive and have been specifically reviewed elsewhere [112]. Some potential routes for solid by-product carbon vary, as shown in Fig. 11. Solid carbon can be used as a catalyst for industrial-scale electrolysis, soil amendments, raw materials for building and construction, and feed for valuable chemical syntheses.

Evaluating the quality of the hydrogen produced from catalytic hydrocarbon decomposition is necessary. Experimental evidence has indicated that hydrogen volumetric and gravimetric densities from the microwave-assisted, Fe-catalysed decomposition of diesel are 71 and 8.6 kg-hydrogen/kg-feed fuel, respectively, which outperform the criteria (70 and 7.5 kg-hydrogen/kg-feed fuel, respectively) set by the United States Department of Energy in 2015 [25]. Therefore, the catalytic hydrocarbon decomposition of fossil fuels is a promising carbon-free, green method for fuel cell-powered transportation and energy storage. More effort (e.g., artificial intelligence [113] and economic analysis) is required to optimize the catalytic process, lower capital and operational costs, and scale-up from an engineering viewpoint for future practical applications. Further studies are required to reveal the economic implications of novel green hydrogen and its scaled-up production and infrastructure.

## 5 Conclusion

Fossil fuels are currently the largest source of short-term hydrogen production. For the first time, this review analyses the available solutions for fossil fuel dehydrogenation by reviewing fossil gas, liquid, solid, and hydrogen production routes with fossil fuel feedstock. amongst the various types of fossil fuels, fossil gas is the best feedstock for all currently used hydrogen production approaches, while fossil minerals are the least preferred owing to their complex compositions of kerogen, organic matter, and mixed fossil fluids. From the perspective of fluid properties, light liquid hydrocarbons perform better than heavy fluids in the dehydrogenation process. However, fossil liquids with a high carbon number perform better. The order of preference of fossil fuels for hydrogen production is fossil gas > heavy fossil liquid > light fossil liquid > fossil minerals.

Hydrocarbon reforming and pyrolysis are still widely used for the hydrogenation of fossil fuel feedstock. However, most hydrocarbon reforming and pyrolysis processes require high capital and operational costs, involve complex procedures, and most importantly, are not carbon-free. Therefore, the thermocatalytic decomposition of hydrocarbons is proposed because it outperforms most other hydrocarbon reforming and pyrolysis processes owing to its energy efficiency, economic effectiveness, and environmental friendliness. The performance of the thermocatalytic process is affected by the selection of catalysts and the activation energy source. Although recent studies have demonstrated that carbon-based catalysts perform better than metal-based catalysts, the metal-based catalysts are still commonly accepted for practical use because of their high activity and easy access. However, there is no consensus on the selection of the most active metals for hydrocarbon decomposition. As for the activation energy source, electromagnetic radiation such as microwaves, which can be directly transferred through molecular interactions, outperforms conventional thermal methods through heat diffusion and convection. By reviewing the resources and technologies of fossil fuel dehydrogenation, this review explores a new mode of “green hydrogen” and confirms that fossil fuels could be absolutely decarbonized, not only from the up-stream exploration and production stages, but also at the end-consuming stage through the conversion to hydrogen. This review reveals that fossil fuels can be utilized in a completely carbon-free manner and provides technical support for future fossil fuel dehydrogenation and energy decarbonization from academic research, industrial practice, and governmental perspectives.

## Declaration of competing interest

The authors declare that they have no conflicts of interest in this work.
